# Enhanced Region Growing for Brain Tumor MR Image Segmentation

**DOI:** 10.3390/jimaging7020022

**Published:** 2021-02-01

**Authors:** Erena Siyoum Biratu, Friedhelm Schwenker, Taye Girma Debelee, Samuel Rahimeto Kebede, Worku Gachena Negera, Hasset Tamirat Molla

**Affiliations:** 1 College of Electrical and Mechanical Engineering, Addis Ababa Science and Technology University, Addis Ababa 120611, Ethiopia; iranasiyoum@gmail.com; 2 Institute of Neural Information Processing, Ulm University, 89081 Ulm, Germany; friedhelm.schwenker@uni-ulm.de; 3 Artificial Intelligence Center, Addis Ababa 40782, Ethiopia; samuelrahimeto@gmail.com (S.R.K.); worku.gachena2@gmail.com (W.G.N.); 4 Department of Electrical and Computer Engineering, Debreberhan University, Debre Berhan 445, Ethiopia; 5 College of Natural and Computational Science, Addis Ababa University, Addis Ababa 1176, Ethiopia; hasset.tamirat@aau.edu.et

**Keywords:** brain MRI image, tumor region, skull stripping, region growing, U-Net, BRATS dataset

## Abstract

A brain tumor is one of the foremost reasons for the rise in mortality among children and adults. A brain tumor is a mass of tissue that propagates out of control of the normal forces that regulate growth inside the brain. A brain tumor appears when one type of cell changes from its normal characteristics and grows and multiplies abnormally. The unusual growth of cells within the brain or inside the skull, which can be cancerous or non-cancerous has been the reason for the death of adults in developed countries and children in under developing countries like Ethiopia. The studies have shown that the region growing algorithm initializes the seed point either manually or semi-manually which as a result affects the segmentation result. However, in this paper, we proposed an enhanced region-growing algorithm for the automatic seed point initialization. The proposed approach’s performance was compared with the state-of-the-art deep learning algorithms using the common dataset, BRATS2015. In the proposed approach, we applied a thresholding technique to strip the skull from each input brain image. After the skull is stripped the brain image is divided into 8 blocks. Then, for each block, we computed the mean intensities and from which the five blocks with maximum mean intensities were selected out of the eight blocks. Next, the five maximum mean intensities were used as a seed point for the region growing algorithm separately and obtained five different regions of interest (ROIs) for each skull stripped input brain image. The five ROIs generated using the proposed approach were evaluated using dice similarity score (DSS), intersection over union (IoU), and accuracy (Acc) against the ground truth (GT), and the best region of interest is selected as a final ROI. Finally, the final ROI was compared with different state-of-the-art deep learning algorithms and region-based segmentation algorithms in terms of DSS. Our proposed approach was validated in three different experimental setups. In the first experimental setup where 15 randomly selected brain images were used for testing and achieved a DSS value of 0.89. In the second and third experimental setups, the proposed approach scored a DSS value of 0.90 and 0.80 for 12 randomly selected and 800 brain images respectively. The average DSS value for the three experimental setups was 0.86.

## 1. Introduction

Cancer is a critical health problem with a very high mortality rate in the world. But we can prevent deaths and illnesses from cancer if we can diagnose it earlier. Globally the mean five-year survival rate of cancer patients has increased from 49% to 67%. The main reason behind this improvement is the rapid growth in diagnostic and treatment techniques [1]. A brain tumor is one of the deadliest cancers among children and adults. A brain tumor is an abnormal mass of brain tissue that grows out of the control of the normal forces that regulate growth inside the skull. These unusual growths can be cancerous or non-cancerous [2]. There are many pieces of research carried out in the past few decades on a brain tumor, but it remained to be one of the major causes among much common type of cancers for the death of people in the entire world [3].

We can classify brain tumors as primary brain tumors and secondary brain tumors depending on the point of origin. Primary brain tumors originate from the brain tissues, whereas secondary tumors originate elsewhere and spread to the brain via hematogenous or lymphatic route. We can categorize brain tumors in terms of severity as benign and malignant [4]:

- Benign brain tumors are those that grow slowly and do not metastasize or spread to other body organs and often can be removed and hence are less destructive or curable. They can still cause problems since they can grow big and press on sensitive areas of the brain (the so-called mass effect). Depending on their location, they can be life-threatening.
- Malignant brain tumors are those with cancerous cells. The rate of growth is fast ranging from months to a few years. Unlike other malignancies, malignant brain tumors rarely spread to other body parts due to the tight junction in the brain and spinal cord.

### Brain Tumor Imaging Technologies

Medical imaging technologies revolutionized medical diagnosis over the last 40 years allowing doctors to detect tumors earlier and improve the prognosis by visualizing tissue structures [5]. The most common imaging modalities for the detection of brain tumors include computed tomography (CT), magnetic resonance imaging (MRI), and positron emission tomography (PET) [5]. MRI is the most commonly used system to diagnose brain tumors since it presents accurate details about the investigated tumor and has little risk to radiations. Additionally, it is capable of differentiating soft tissue with high resolution and is more sensitive in detecting and visualizing subtle changes in tissue density and the physiological alternations associated with the tumor [6,7,8,9]. Usually, one imaging modality is used in the diagnosis of brain tumors. But in some cases, more than one imaging modality might be advantageous in the diagnosis of brain tumors using medical image registration. Rundo et al. [10] explored the use of medical registration, which is a process of combining information from different imaging modalities into single data. These fusions usually require optimization of similarity between the different modality input images. CNN based optimization for medical image registration was performed in [11].

MRI is a non-invasive imaging technique that produces three-dimensional anatomical images by measuring the energy released when a proton changes its polarity after it was altered using a strong magnetic field. MRIs are sain the detection of abnormalities in the soft tissues.

MRI images can be taken in many ways [12]. The most common and widely used modalities include:

- T1-weighted: by measuring the time required for the magnetic vector to return to its resting state(T1-relaxation time)
- T2-weighted: by measuring the time required for the axial spin to return to its resting state (T2-relaxation time).
- Fluid-attenuated inversion recovery(T2-FLAIR): which is T2 weighted by suppressing cerebrospinal fluid(CSF).

T1, especially with the addition of contrast(Gadolinium), is effective in the detection of new lesions, whereas T2 and Flair are effective in defining high-grade glial neoplasm(glioma) and surrounding edema. Flair performs better in defining the actual volume of the neoplasm [13]. In this paper, we considered Flair images since they are effective in the detection of Gliomas (such as glioblastoma, astrocytomas, oligodendrogliomas, and ependymomas), that makeup 81% of malignant brain tumors in adults [14].

## 2. Related Works

Since medical images contain artifacts such as tags, noises, and other body parts that are not the area of interest, they needed to be removed [15]. Then, segmentation tasks are performed to extract the region of interest for the detection and classification step. Recently, deep-learning based methods tried to combine both segmentation and classification of medical images in one process. Brain tumor segmentation can be categorized into region-based and deep-learning-based segmentations. From region-based segmentation algorithms, we will be addressing clustering, region growing, fuzzy means segmentation algorithms. And, from deep learning U-Net has been addressed.

### 2.1. Region-Based Brain Tumor Segmentation

A lot of researches have been carried out in the area of segmentation for medical images like breast cancer and brain tumor using various segmentation methods [16]. However, the complexity and large variations of the tissue structure and indistinguishable boundaries between regions of the human brain tissues made the brain tumor segmentation a challenging task [17]. In the past few years, different brain image segmentation approaches have been developed for MRI images and evaluated using different evaluation parameters.

One of the most common, easiest, and fastest algorithm for image segmentation is thresholding. The thresholding technique is based on one or more intensity threshold values where these values are compared with pixel intensities. Thresholding performs well when there is homogeneous intensity in the image. However, applying the thresholding segmentation algorithm to brain tumor segmentation is not recommended because of two reasons: optimal threshold selection is not an easy task, and intensity in the brain tumor is not homogeneous [18]. These problems have been tried to be addressed using image enhancement techniques for clearly differentiating between tissue regions on MRI scans. Rundo et al. [19] proposed a novel medical image enhancement technique called medGA, which is a pre-processing technique based on the genetic algorithm. But medGA needs a user input for the ROI from the MRI slices. Acharya and Kumar [20] proposed a particle-swarm-based contrast enhancement technique for brain MRI images. They have compared the proposed algorithm with other contrast enhancement techniques. But they didn’t show its performance when it is applied as a pre-processing for segmentation using a thresholding technique.

The other commonly used segmentation algorithm in medical images is the watershed algorithm. The working principle behind the watershed segmentation algorithm is similar to the water flooding in the rigged landscape [18]. The watershed algorithm can accurately segment multiple regions at the same time with complete contour for each section. But, the watershed segmentation algorithm suffers from over-segmentation [21].

The region growing algorithm is one of the most successful approaches for brain tumor segmentation. This approach mainly extracts regions with similar pixels [18]. The region-growing algorithm’s performance is highly dependent on the initial seed point selection and the type of similarity measure used between neighboring pixels. However, in most cases selecting an optimal seed point is made manually as presented in Table 1 and a challenging task besides its higher computational cost [18].

Salman et al. [22] and Sarathi et al. [23] stated that region growing segmentation algorithm has shown better performance for brain tumor segmentation to generate ROI. However, Salman et al. [22] in their work manually selected the initial point as the seed for the region growing algorithm-based approach that they proposed to get ROI. Thiruvenkadam [24] explained that manual seed point selection is the most important step for region growing based brain tumor segmentation.

Cui et al. [17] fused two MRI images (MRI-FLAIR and MRI-T2) for generating initial seed points for the region growing algorithm. They automatically select seed points but the overall algorithm is not consistent. The inconsistency comes from the fact that seed points are selected randomly from a set of potential seed points generated by calculating seeds’ probability of belonging to a tumor region.

Sarathi et al. [23] proposed a wavelet features based region growing segmentation algorithm for an original 256 × 256 T1-weighted enhanced MRI image. For the selection of seed points, they first convolved the 64 × 64 kernel with the 64 × 64 preprocessed brain images and followed by wavelet feature extraction. Then significant wavelet feature points were used alternatively as a potential initial seed until the best ROI is extracted. In this paper, mean, variance, standard deviation, and entropy were used as similarity properties to include or exclude the neighboring pixels to the seed point. The experimental result showed that the proposed approach gave better performance results with minimum computational time.

In [25] the intensity values of brain tissue from its different regions were considered to decide the selection of the seed points. However, brain map structure and intensity information need to be known in advance. Therefore, to gain detailed information on the brain images, multi-modal images were preferred, and hence in this work Ho et al. [25] used a fusion of multi-modal images to select the initial seed automatically.

Bauer et al. [26] used a soft-margin SVM classifier for the segmentation of brain tumors hierarchically by classifying MRI voxels. 28 features were extracted from the voxel intensity and first-order textures extracted from patches around the voxel. Conditional Random Fields(CRF) regularization was applied to introduces spatial constraints to the SVM classifier since considers each voxel is independent. The proposed algorithm achieved a DSS of 0.84. They didn’t specify the size of patches taken around the voxels when extracting texture features. There was no comparison performed with state-of-the-art algorithms.

Rundo et al. [27] used Fuzzy C-Means(FCM) based segmentation algorithms to segment the whole tumor volume using their gross tumor volume (GTV) segmentation in the first step and extract the necrosis volume from the gross tumor volume in the second step. But the proposed algorithm needs human intervention for the GTV algorithm.

### 2.2. Deep Learning-Based Brain Tumor Segmentations

Deep learning has been applied for the classification and segmentation of medical images previously [28,29,30,31,32]. Different versions of CNNs were used for the segmentation of brain tumors from MRI scans.

Li et al. [33], applied generative adversarial networks(GANs) to augment brain datasets by generating realistic paired data. The proposed method can augment n data pairs into n 2-n data. Their data augmentation technique was used to train and test different deep learning-based segmentation techniques using the BRATS2017 dataset. The best performer, the U-net algorithm, achieved a DSS of 0.754 when using the original dataset but this performance was improved to 0.765 in the case of whole tumor segmentation. The network architecture of U-Net is symmetric and composed of Encoder and decoder. The encoder is used to extract features from the input images and decoder constructs segmentation from the extracted features in Encoder [34]. U-Net became the most popular semantic segmentation in medical imaging [34]. In this paper, U-Net was implemented for comparing the performances of our proposed model.

Rundo et al. [35] modified the original U-Net architecture by adding squeeze-excitation (SE) blocks in every skip connection. They proposed two architectures, first only the encoder block output was feed to SE blocks at the skip connection. Another architecture was modifying each skip connection by adding SE blocks at every encoder and decoder block and combine the outputs to modify the original skip connection. The SE blocks are designed to model interdependencies between channels and increases the model generalization capabilities when trained using different datasets. The datasets consisted of prostate MRI scans for zonal segmentation collected from various institutions. The SE block’s ability to adaptive feature recalibration significantly improves the performances of the U-net architecture, when trained across different datasets.

## 3. Materials and Methods

**Figure 1:**
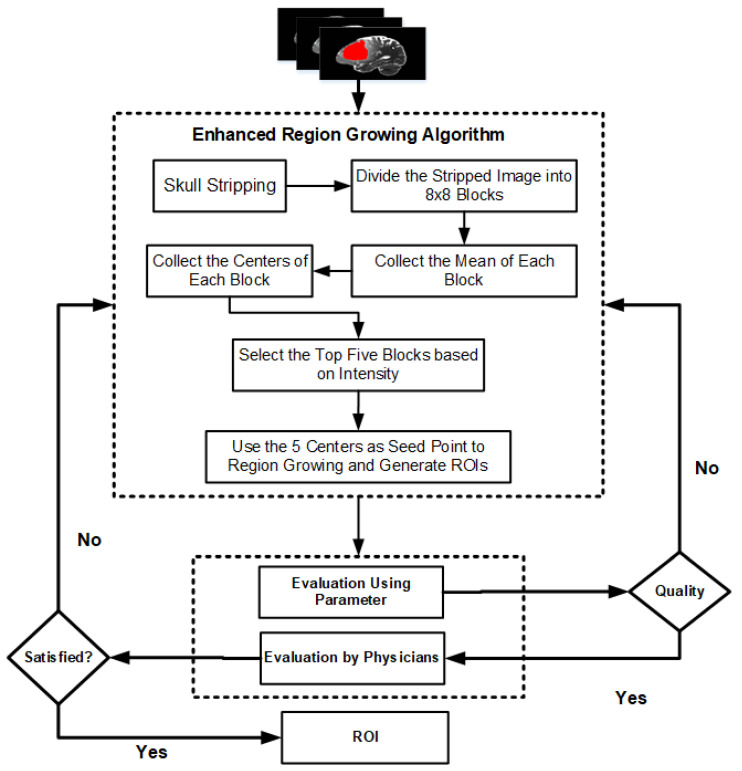
Flowchart of the proposed region growing algorithm. In this approach the segmentation result is evaluated both by evaluation parameters and Physicians/Radiologists.

Figure 1 presents the flowchart of the proposed enhanced region-growing algorithm for brain tumor segmentation. Raw MRI images usually have different artifacts and non-brain parts that affect the segmentation quality and hence a preprocessing step should be applied before segmentation algorithms. The enhanced region-growing algorithm is applied to generate candidate brain tumor regions. The detail methods used in this paper is presented in Section 3.1 through Section 3.4.

### 3.1. Dataset

The image dataset used in this paper contains multimodal MRI scans of patients with gliomas (54 LGGs and 132 HGGs). It was used for the multimodal Brain Tumor Segmentation (BRATS) 2015 challenge, from the Virtual Skeleton Database (VSD) [36]. Specifically, these image datasets were a combination of the training set (10 LGGs and 20 HGGs) used in the BRATS 2013 challenge [37], as well as 44 LGG and 112 HGG scans provided from the National Institutes of Health (NIH) Cancer Imaging Archive (TCIA). The data of each patient consisted of native and contrast-enhanced (CE) T1-weighted, as well as T2-weighted and T2 Fluid-attenuated inversion recovery (FLAIR) MRI volumes.

In the dataset, the ground truth (GT) was included for training the segmentation model and qualitative evaluation. Specifically, the data from BRATS 2013 were manually annotated, whereas data from TCIA were automatically annotated by fusing the approved by experts results of the segmentation algorithms that ranked high in the BRATS 2012 and 2013 challenges [37]. The GT segmentations comprise the enhancing part of the tumor (ET), the tumor core (TC), which is described by the union of necrotic, non-enhancing, and enhancing parts of the tumor, and the whole tumor (WT), which is the union of the TC and the peritumoral edematous region.

### 3.2. Preprocessing

In digital image processing preprocessing plays an important role in smoothing and normalizing the MRI images [38]. Performing preprocessing suppresses the impact of dark parts in the borders of the brain images [38].

The BRATS2015 dataset is available in a preprocessed format in which unwanted parts are removed. But, preprocessing is essential for raw MRI data. Skull Stripping is one of the popular pre-processing techniques that remove the skull from brain image. The surroundings of a brain are termed as a skull. The skull stripping is the process of eradicating the tissues that are not cerebral. It is difficult to distinguish non-cerebral and the intra-cranial tissues because of their homogeneity in intensities [39]. In brain tumor segmentation, stripping the skull and other non-brain parts is a crucial step to be accomplished but it is a challenging task [40]. The challenge arises from large anatomical variability among brains, different acquisition methods of brain images, and the existence of artifacts on brain images. These are some of the reasons among many that boost the challenge to design a robust algorithm [40]. Segonne et al. [40] proposed a hybrid approach that was used to strip the skull where they combined the watershed algorithm and deformable surface model.

**Figure 2:**
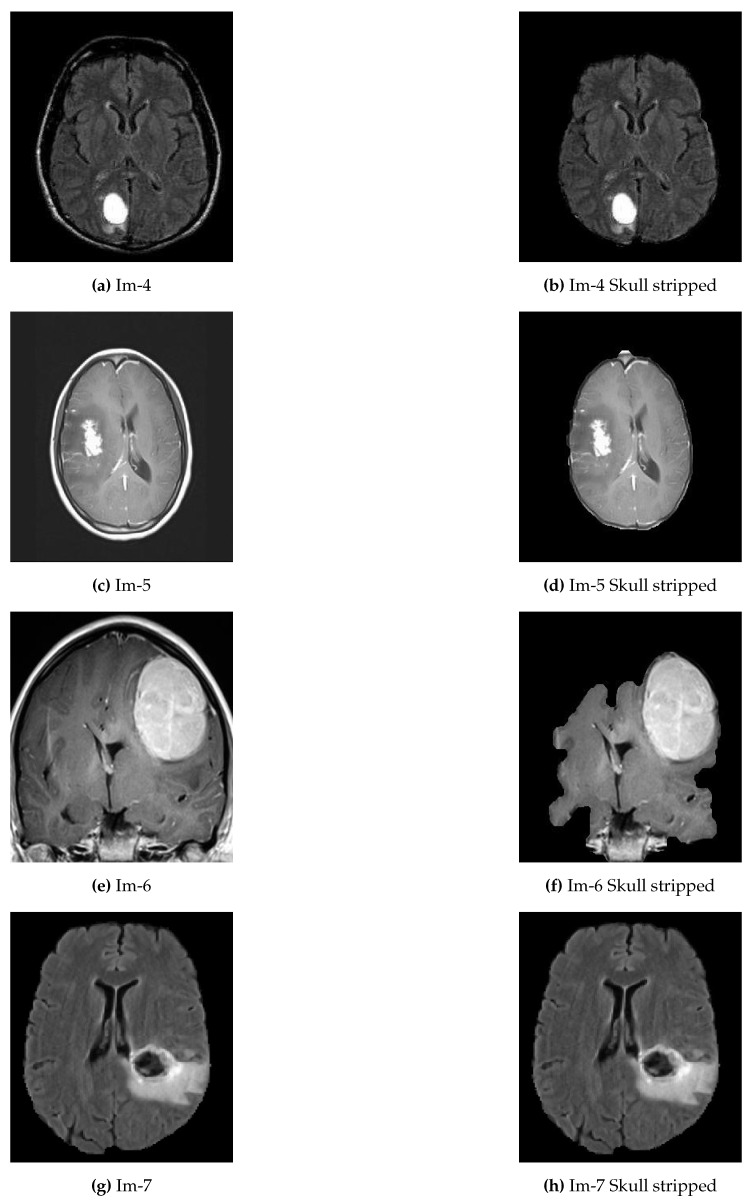
Examples of original abnormal brain tumor images before and after skull removed. (**a**,**c**,**e**,**g**) represent original brain images with skull; (**b**,**d**,**f**,**h**) represent the skull removed original brain images.

In the proposed approach, we applied thresholding and morphological operation for preprocessing (see Algorithm 1). Since the MRI images in the local dataset are images with three color channels, it was changed into a grayscale image before the preprocessing. Otsu’s thresholding technique was employed to determine the threshold between the background and the tissue regions. By thresholding, the largest binary object extracts the brain and removes the skull and other tags from the image. Some examples of skull removal algorithm are presented in Figure 2.
**Algorithm 1** Skull Stripping1:**input:** gray scale image, im2:Calculate Otsu’s Threshold T←graythresh(im)3:Threshold the image BW←im2bw(im,T)4:Open the binary image using a disk structuring Seed BW←imopen(BW,se)5:Dilate the binary image BW←imdilate(BW,se)6:Select the largest binary image BW←largest_blob(BW)7:Dilate the binary image BW←imclose(BW,se)8:Fill holes on the binary image BW←imfill(BW,se)9:Remove the skull stripped←im(!BW)=010:**return** stripped

### 3.3. Enhanced Region-Growing Approach

The proposed enhanced region-growing based approach that automatically detect the abnormality region and extract the ROI for each brain image is presented in Algorithm 2. This approach is the main contribution of the paper. The role of Algorithm 1 is to strip the skull of the input original brain image. Then, the skull stripped brain image is divided into 32 blocks or patches of size 8×8. For each block${}_{i}$, the average (mean) intensity was computed as indicated in Equation (1):

(1) $$AvgI_{i=1:32}=\frac{\sum_{j=1}^{8}\sum_{k=1}^{8}I_{jk}}{64}$$

**Figure 3:**
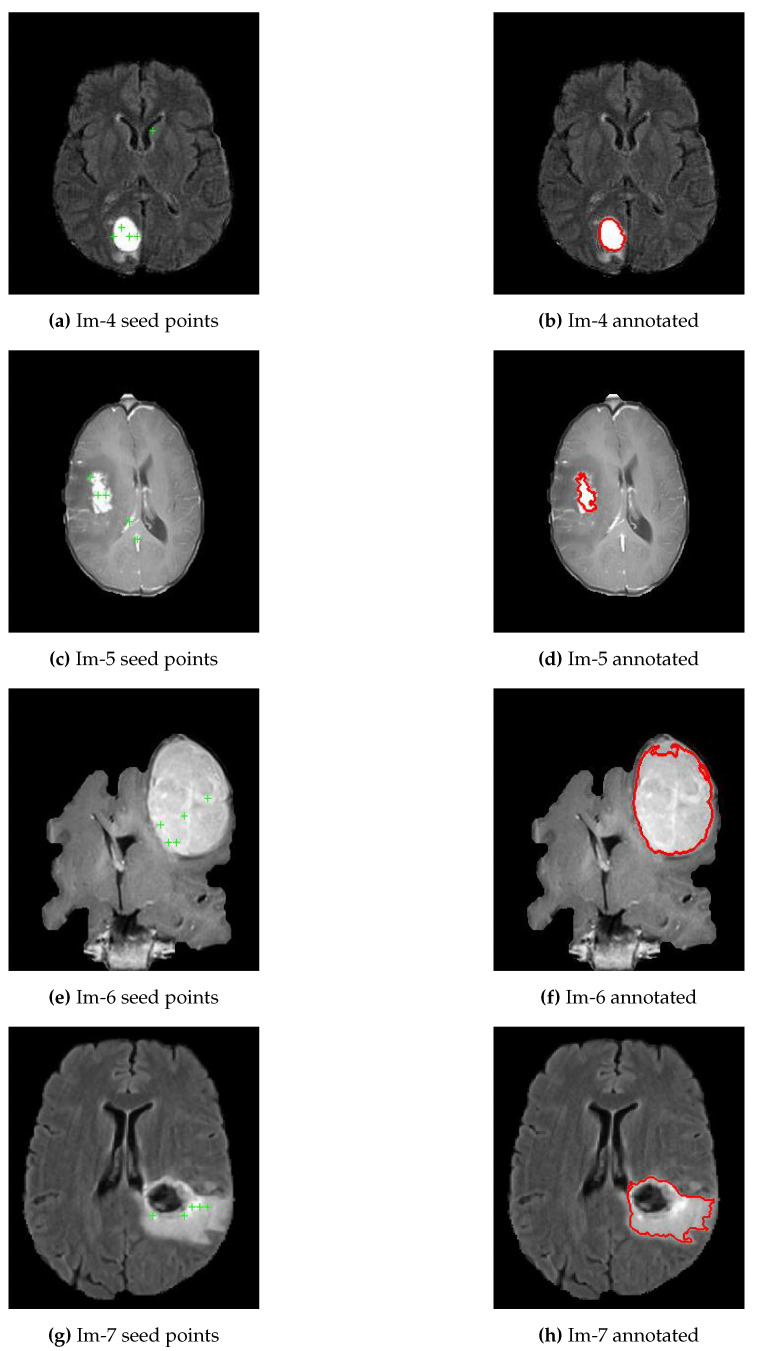
Generated possible seed points and annotations using proposed approach. (**a**,**c**,**e**,**g**) represent a skull removed original brain images with five potential seed points for brain images; (**b**,**d**,**f**,**h**) represent the best ROIs of each respective brain images.

As presented in Algorithm 2, line 6 and Equation (1), the mean intensities for each of the 32 blocks were computed and selected only the top five brightest pixels as potential candidates to use as seed points for the region-growing segmentation algorithm, refer Figure 3a,c,e,g. Line 12 to 14 of Algorithm 2 presented the five ROIs generated by region-growing segmentation algorithm, and then compared the results against the ground truth using evaluation parameters to select the best ROI as a final segmentation output, see Figure 3b,d,f,h. The region-growing segmentation algorithm’s threshold point is determined experimentally to be 0.1 since most of the tumor regions appear homogeneous. However, some of the inhomogeneities parts were accommodated with fill hole operations as shown in Figure 3h. In this particular brain image, the tumor core appears black and our algorithm might detect only the boundaries. But, for such cases, we applied the fill holes operations to include the core of the tumor.

**Algorithm 2** Enhanced Region Growing Segmentation for Brain Tumor Segmentation
1:**input:** skull stripped image, im2:Resize the Image       im←imresize(im,[256,256])3:iterate through each 8×8 block4:
**for** i=1:8:256 **do**
5:
** for** j=1:8:256 **do**
6:  Collect the mean of each block         mIs←mean(im(i:i+7,j:j+7)7:  Collect the centers of each block  cBs←[i+3,j+3]
8: **end for**
9:  **end for**
10:Select top 5 blocks based on the intensity        [ind,vals]=max(mIs,5)        seeds=cBs(ind)11:
**return** seeds
12:
**for** m=1:5 **do**
13: ROI${}_{m}$= Region-growing(seed${}_{m}$)14:
**end for**
15:Compare each ROI${}_{m}$ against GT using evaluation parameters for m=1:516:Select the best ROI as a final segmentation output.


### 3.4. Evaluation Approach

The most common parameters to be used to evaluate the performance of segmentation algorithms are DSS, Similarity Index (SI), Extra Fraction (EF), Overlap Fraction (OF), Jaccard Similarity (JSI), accuracy (Acc), sensitivity (Sn), specificity (Sp), computation cost, Root Mean Squared Error (RMSE) and intersection over union (IoU). JSI is similar with IoU and Sp is similar with SI.

Consider True Positive (TP) as the number of tumor region pixels correctly identified and classified, False Positive (FP) as the number of normal region pixels in the input image identified as tumor region, False Negative (FN) as the number of tumor region pixels left undetected or misclassified, and True Negative (TN) as the number of normal region pixels in the input region identified as the normal region.

#### 3.4.1. Extra Fraction (EF)

Extra fraction refers to the number of pixels being falsely detected as a tumor region. A minimum extra fraction value means a better segmentation result [41].

(2) $$EF=\frac{FP}{TP+FN}$$

#### 3.4.2. Overlap Fraction (OF)

Overlap fraction or sensitivity value refers to the number of images segmented and classified correctly [41]. Specifically, overlap fraction refers to the tumor region being correctly identified.

(3) $$OF=\frac{TP}{TP+FN}$$

#### 3.4.3. Dice Similarity Score (DSS)

It measures the spatial overlap between the original image and the segmented target region.

(4) $$DSS=\frac{TP}{\frac{1}{2}\left(2TP+FP+FN\right)}$$

Besides, we have involved the radiologist to evaluate the final ROIs obtained using the proposed approach for randomly selected brain images to validate our proposed approach qualitatively.

## 4. Experimental Results and Discussion

The first experimental result was the skull stripped brain images as indicated in Figure 2 where Figure 2a,c,e,g were the original brain images of size 256×256 and Figure 2b,d,f,h were the skull stripped brain images. Then, as presented in Equation (1), we generated 32 average intensities for each skull stripped brain images and selected the five top average intensities for each image and used as potential initial seed points for region growing algorithm as indicated in Figure 3a,c,e,g. Using the five selected initial seed points for each image, we generated five different ROIs and compared against the respective GT and selected the best ROI as presented in Figure 3b,d,f,h.

To validate the proposed approach, we designed three different experimental setups for analysis. In our first experiment, we randomly selected 15 brain images from the BRATS2015 dataset. In the second experiment, we again randomly selected 12 brain images from the same dataset and finally, in the third experimental setup, we used 800 brain images from the same dataset used in the previous two experimental setups.

In all the three experimental setups, the performance of the proposed approach was evaluated in terms of Acc, IoU, DSS, Sn, Sp, EF, OF, and PSNR. In most cases, especially the deep learning algorithms use DSS to evaluate the segmentation algorithms. The highest value of Acc, IoU, DSS, Sn, Sp, OF and PSNR indicate the highest performance whereas the lowest value of EF indicates poor performance.

In the first experimental setup, 15 brain images were used for experimental analysis, and for each image, the corresponding Acc, IoU, DSS, Sn, Sp, OF, EF, and PSNR were computed as indicated in Table 2. The average value of Acc, IoU, DSS, Sn, Sp, OF, EF, and PSNR for the 15 brain images were used to compare the performance of the proposed approach with that of modified adaptive K-means and U-Net.

Table 2 indicates that the proposed algorithm outperformed modified adaptive K-means, and U-Net in terms of an average value of Acc, IoU, DSS, EF, and PSNR. However, it achieved a lower average value of Sn, Sp, and OF. The lower average value of Sn, Sp, and OF is achieved because of the least value of respective parameters for images 14 and 15. However, still, the U-Net and MAKM have an insignificant higher performance than the proposed approach. In the case of OF and Sn, U-Net achieved 4% and MAKM achieved 1% higher than the proposed approach. In the case of Sp, both the U-Net and MAKM are 1% higher than the proposed approach.

Table 3 presented the comparison of the proposed approach, MAKAM and U-Net for the 12 randomly selected brain images from BRATS2015. The proposed approach scored a higher value of Acc, IoU, DSS, Sp, EF, and PSNR but a lower value of Sn and OF compared to MAKM and U-Net. The value of Acc, IoU, DSS, Sp, EF, and PSNR were 99.1%, 0.82, 0.90, 99.7%, 0.06, and 163.89 respectively whereas the value of Sn and OF were 89.1% and 0.89 respectively. U-Net achieved a higher value for both Sn and OF compared to MAKM and the proposed approach where performance difference was limited to nearly to 2%.

Table 4 presented the experimental results of the proposed approach for 800 brain images and compared them with the performance of MAKM and U-Net. The experimental results showed in Table 4 indicated that the proposed approach scored a higher value of Acc, IoU, DSS, Sp, EF, and PSNR but a lower value of Sn and OF compared to MAKM and U-Net. The value of Acc, IoU, DSS, Sp, EF, and PSNR was 98.72%, 0.67, 0.80, 99.8%, 0.06, and 157.0 respectively whereas the value of Sn and OF were 90.7% and 0.91 respectively. The higher value of Sn and OF were scored by U-Net.

Table 5 presented the achieved state-of-the-art deep learning algorithms’ results on the BRATS2015 dataset and compared with the scored performance of the proposed approach for three different experimental setups/cases. The experimental results achieved were the DSS value of 0.89, 0.90, and 0.80 for case-1, case-2, and case-3 respectively. The average DSS value of the three experimental setups was 0.86. In this paper, no classifier was applied for final segmentation but the enhanced region growing algorithm was effective in generating candidate regions of interest. We did choose the best ROI against GT from the generated ROIs to compare with the other methods. From the experimental results, we saw that the proposed approach can generate the best ROI in most of the test cases. But still, a classifier should be trained by extracting features from the abnormal ROIs for making the algorithm to detect and determine the tumor type.

**Figure 4:**
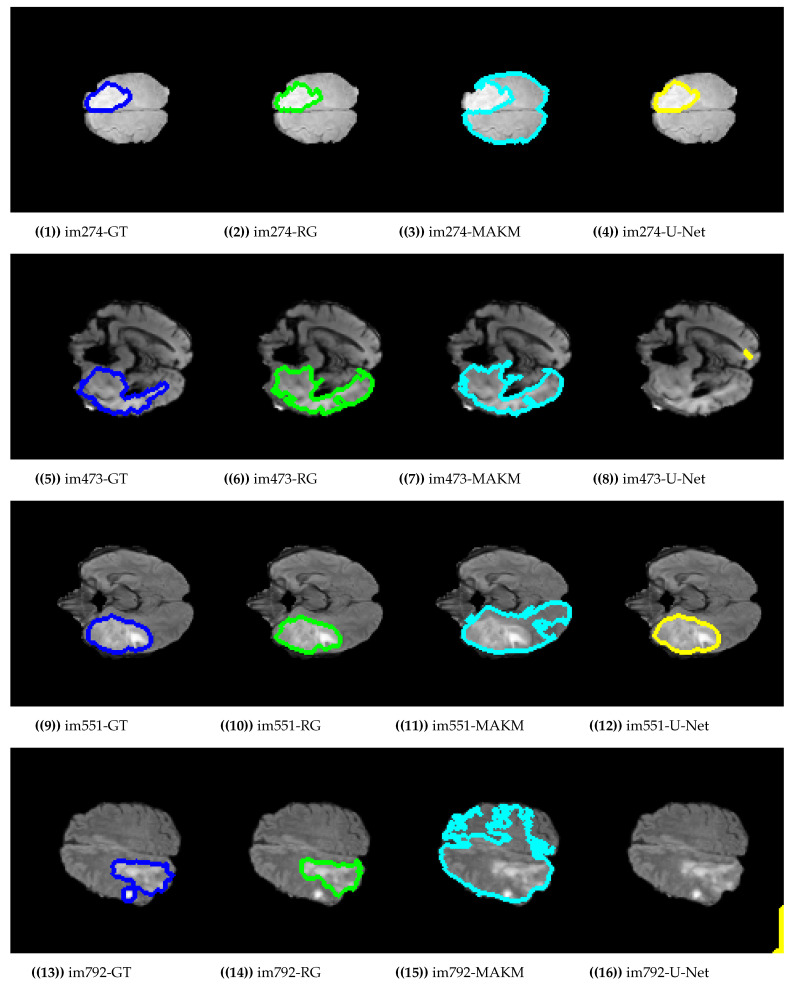

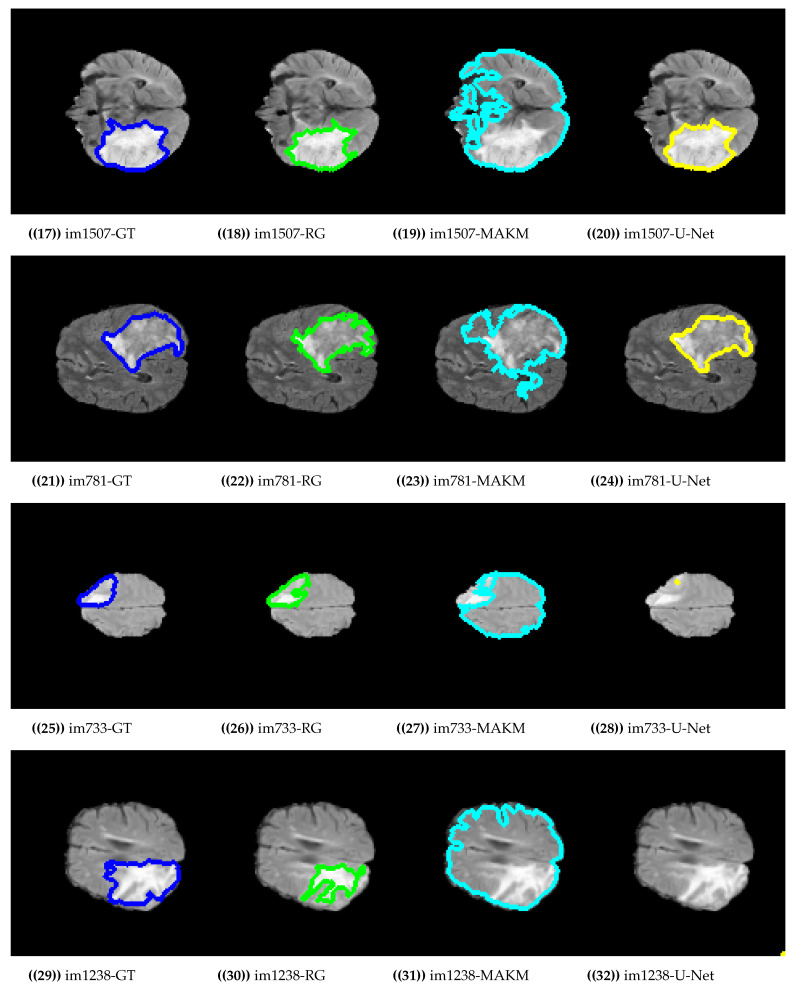
Segmentation results on BRATS2015 dataset.

Figure 4 presented the segmentation results of the proposed algorithm, MAKM, and U-Net in terms of ROIs and their respective ground truths. For im274, im473, im551, im1507, im781, and im733 the proposed approach achieved ROIs which were almost the same as their respective ground truths (GTs). The proposed approach resulted in under-segmentation for im792 and im1238 as indicated in Figure 4. For the case of U-Net, the good segmentation results were observed only for im274, im551, im1507, and im781 and unable to detect the tumor region for im473, im792, im733, and im1238. In the case of MAKAM, over-segmentation results were achieved in almost all randomly selected brain images except for im274 where it detected the normal brain image part as abnormal.

For comparison purposes, we evaluated the performance of the proposed approach with MAKM and U-Net. MAKM [46] is a modified version of the adaptive k-means algorithm proposed by Debelee et al. The performance of the proposed approach was by far better than the MAKM algorithm that mainly proposed for detection of cancer on mammographic images. For the case of U-Net, we first trained the U-Net architecture from the scratch using 16000 slices extracted from MRI scans of 200 patients obtained from the BRATS2015 datasets, with 80 slices per patient (slice 50 to 130). The 200 patients were affected by the fast-growing and rapidly spreading tumors called High-Grade Glioma. The training was performed for 50 epochs until we got no significant improvements. Since the BRATS2015 datasets consisted of MRI scans with much of the preprocessing (such as tag removal and skull stripping) performed, we just applied intensity normalization before the training. We used DSS as the loss function in the training process, for the training of a nine-layer U-net architecture described in [47]. This architecture has an additional batch normalization after each convolutional layer and for evaluation purposes, we randomly selected 15 brain images for the testing after model validation and the testing DSS score value was less by 14% compared with the proposed approach.

Finally, we compared the performance of the proposed approach with the U-Net and its variants based on the BRATS2015 dataset. Daimary et al. [42] and Zhou et al. proposed a U-Net variant architecture and scored a DSS value of 0.73 and 0.87 respectively which was less than what the proposed approach scored. Havaei et al. [43] have evaluated their approach using the BRATS2015 dataset and achieved 0.88, 0.79, and 0.73 for three modalities, whole, core and enhanced respectively.

## 5. Conclusions

The brain tumor is one of the major cancer types which has been a reason for the higher death rate in the entire world. To combat that a significant number of medical image analysis-based research works have been carried for different types of cancer detection and classification using deep learning and conventional/shallow machine learning approach. Shallow machine learning is usually applied in combination with digital image processing techniques for image-based analysis. In this article, we modified the existing and popular region-growing segmentation algorithm to detect the abnormality region on brain images. The main challenge of the region-growing algorithm is seed point initialization to get the best ROI for any input brain images. In the proposed approach the seed point initialization was made to be automatically generated for any input brain images and tested on the BRATS2015 dataset in three different experimental setups. The experimental result of our approach was compared with MAKM, U-Net architecture, and its variant for brain tumor detection and segmentation. From the experimental result, we have seen that the proposed algorithm can detect brain tumor locations and extract the best ROIs. The results of the proposed method achieved higher performance than modified adaptive k-means. Almost all U-Net architecture and its variants have scored lesser DSS Value for the BRATS2015 brain tumor image dataset. However, in most of the cases, the U-Net either over-segments or missed the tumor region of the brain MRI images. The proposed approach has a problem in thresholding point selection for the region-growing algorithm and was left for future work.

## Figures and Tables

**Table 1:** Related Work in region growing seed selection and growth criteria.

Authors and Citation	Seed Selection	RG Criteria
Salman et al., 2006 [22]	Manual	Texture
Sarathi et al., 2013 [23]	Automatic	variance, Entropy
Thiruvenkadam, 2015 [24]	Manual	-
Ho et al., 2016 [25]	Automatic	Intensity
Cui et al., 2019 [17]	Semi-automatic	Intensity & Spatial Texture

**Table 2:** Performance comparison of RG with MAKM and U-Net for 15 randomly selected brain images from BRATS2015 Dataset.

Metric	Algorithm	im01	im02	im03	im04	im05	im06	im07	im08	im09	im10	im11	im12	im13	im14	im15	Avg
	RG	100	100	100	100	99	99	99	99	99	99	100	99	99	88	94	98
Acc (%)	MAKM	99	99	99	82	99	99	99	99	86	86	80	87	99	87	99	93
	U-Net	100	100	100	100	98	98	74	74	99	99	67	99	100	99	92	93
	RG	0.94	0.94	0.94	0.93	0.88	0.88	0.85	0.85	0.85	0.85	0.84	0.83	0.81	0.31	0.04	0.78
IoU	MAKM	0.90	0.79	0.79	0.21	0.86	0.86	0.90	0.90	0.26	0.26	0.06	0.19	0.81	0.34	0.65	0.59
	U-Net	0.94	0.96	0.96	0.93	0.70	0.70	0.16	0.16	0.91	0.91	0.03	0.84	0.93	0.81	0.24	0.68
	RG	0.97	0.97	0.97	0.96	0.93	0.93	0.92	0.92	0.92	0.92	0.91	0.91	0.89	0.47	0.80	0.89
DSS	MAKM	0.95	0.88	0.88	0.35	0.92	0.92	0.95	0.95	0.42	0.42	0.11	0.33	0.90	0.51	0.79	0.68
	U-Net	0.97	0.98	0.98	0.96	0.82	0.82	0.27	0.27	0.95	0.95	0.07	0.92	0.96	0.89	0.39	0.75
	RG	97	95	95	98	88	88	87	87	85	85	85	83	81	100	100	90
Sn (%)	MAKM	91	79	79	100	86	86	96	96	100	100	100	99	85	100	65	91
	U-Net	100	98	98	93	95	95	99	99	100	100	90	100	96	88	65	94
	RG	100	100	100	100	100	100	100	100	100	100	100	100	100	84	00	92
Sp (%)	MAKM	100	100	100	81	100	100	100	100	85	85	79	86	100	86	100	93
	U-Net	100	100	100	100	98	98	73	73	99	99	67	99	100	99	93	93
	RG	0.03	0.02	0.02	0.06	0.00	0.00	0.02	0.02	0.00	0.00	0.01	0.00	0.00	2.27	23.37	1.72
EF	MAKM	0.01	0.00	0.00	3.79	0.00	0.00	0.06	0.06	2.79	2.79	16.11	4.09	0.04	1.92	0.00	2.11
	U-Net	0.05	0.02	0.02	0.00	0.35	0.35	5.23	5.23	0.10	0.10	25.57	0.18	0.03	0.08	1.69	2.60
	RG	0.97	0.95	0.95	0.98	0.88	0.88	0.87	0.87	0.85	0.85	0.85	0.83	0.81	1.00	1.00	0.90
OF	MAKM	0.91	0.79	0.79	1.00	0.86	0.86	0.96	0.96	1.00	1.00	1.00	0.99	0.85	1.00	0.65	0.91
	U-Net	1.00	0.98	0.98	0.93	0.95	0.95	0.99	0.99	1.00	1.00	0.90	1.00	0.96	0.88	0.65	0.94
	RG	72.72	74.40	74.40	72.72	70.22	70.22	69.50	69.50	69.38	69.38	75.09	70.79	68.25	56.31	48.31	68.75
PSNR	MAKM	70.63	69.38	69.38	55.51	69.67	69.67	71.02	71.02	56.66	56.66	55.02	56.88	68.19	57.03	66.53	64.22
	U-Net	72.99	76.49	76.49	72.64	65.12	65.12	54.06	54.06	71.02	71.02	53.00	70.37	72.64	66.70	58.92	66.71

**Table 3:** Performance comparison of RG with MAKM and U-Net for 12 randomly selected brain images from BRATS2015 Dataset.

Metric	Algorithm	im081	im274	im473	im551	im06	im973	im689	im792	im1507	im781	im733	im1238	Avg
	RG	99.6	99.8	97.4	99.6	99.6	99.7	100.0	99.1	98.7	99.2	99.7	96.8	99.1
Acc (%)	MAKM	84.9	89.1	97.2	95.9	85.4	79.7	76.9	87.7	84.3	95.6	90.4	84.6	87.6
	U-NET	99.8	99.8	93.3	99.8	99.8	98.7	99.8	89.2	99.5	99.5	99.1	86.6	97.1
	RG	0.91	0.92	0.62	0.92	0.92	0.94	0.89	0.77	0.80	0.88	0.85	0.47	0.82
IoU	MAKM	0.05	0.01	0.61	0.50	0.23	0.02	0.02	0.23	0.29	0.58	0.04	0.28	0.24
	U-NET	0.95	0.93	0.39	0.94	0.95	0.76	0.61	0.25	0.92	0.93	0.45	0.31	0.70
	RG	0.95	0.96	0.76	0.96	0.96	0.97	0.94	0.87	0.89	0.94	0.92	0.64	0.90
DSS	MAKM	0.09	0.01	0.75	0.67	0.38	0.03	0.03	0.37	0.44	0.74	0.09	0.44	0.34
	U-NET	0.98	0.96	0.56	0.97	0.97	0.86	0.76	0.40	0.96	0.96	0.62	0.47	0.79
	RG	95.3	93.9	83.5	92.1	94.5	96.2	92.2	82.0	79.8	91.2	92.9	46.8	86.7
Sn (%)	MAKM	18.2	3.5	89.1	99.3	100.0	7.4	100.0	96.9	99.4	99.9	25.8	98.5	69.8
	U-NET	98.9	97.7	85.7	98.0	98.8	97.2	60.9	98.3	92.9	95.4	45.2	99.9	89.1
	RG	99.8	100.0	98.2	100.0	99.9	99.9	100.0	99.8	100.0	99.8	99.8	100.0	99.7
Sp (%)	MAKM	87.9	90.9	97.6	95.7	84.7	82.9	76.8	87.4	83.3	95.3	91.6	83.7	88.2
	U-NET	99.8	99.9	93.7	99.8	99.8	98.8	100.0	88.9	99.9	99.8	100.0	85.8	97.2
	RG	0.05	0.02	0.35	0.01	0.03	0.02	0.03	0.06	0.00	0.03	0.09	0.00	0.06
EF	MAKM	0.18	0.03	0.89	0.99	1.00	0.07	1.00	0.97	0.99	1.00	0.26	0.98	0.70
	U-NET	0.99	0.98	0.86	0.98	0.99	0.97	0.61	0.98	0.93	0.95	0.45	1.00	0.89
	RG	0.95	0.94	0.83	0.92	0.94	0.96	0.92	0.82	0.80	0.91	0.93	0.47	0.87
OF	MAKM	0.18	0.03	0.89	0.99	1.00	0.07	1.00	0.97	0.99	1.00	0.26	0.98	0.70
	U-NET	0.99	0.98	0.86	0.98	0.99	0.97	0.61	0.98	0.93	0.95	0.45	1.00	0.89
	RG	165.52	174.19	147.51	167.26	166.43	170.25	188.41	157.89	154.39	159.74	169.80	145.23	163.89
PNSR	MAKM	129.72	132.99	146.42	142.69	130.06	126.75	125.49	131.81	129.36	142.02	134.30	129.55	133.43
	U-NET	172.31	175.68	137.87	170.98	171.49	154.11	175.68	133.12	163.80	164.69	157.43	130.96	159.01

**Table 4:** Performance comparison of RG with MAKM and U-Net for 800 brain images from BRATS2015 Dataset.

Metric	Algorithm	im081	im274	im473	im551	im06	im973	im689	im792	im1507	im781	im733	im1238	im368	…	im551	Ovr_Avg
	RG	99.6	99.8	97.4	99.6	99.6	99.7	100.0	99.1	98.7	99.2	99.7	96.8	95.2	…	97.8	98.72
Acc (%)	MAKM	84.9	89.1	97.2	95.9	85.4	79.7	76.9	87.7	84.3	95.6	90.4	84.6	98.8	…	98.7	88.60
	U-NET	99.8	99.8	93.3	99.8	99.8	98.7	99.8	89.2	99.5	99.5	77.6	86.6	83.8	…	99.8	98.20
	RG	0.91	0.92	0.62	0.92	0.92	0.94	0.89	0.77	0.80	0.88	0.85	0.47	0.28	…	0.77	0.67
IoU	MAKM	0.05	0.01	0.61	0.50	0.23	0.02	0.02	0.23	0.29	0.58	0.04	0.28	0.81	…	0.85	0.34
	U-NET	0.95	0.93	0.39	0.94	0.95	0.76	0.61	0.25	0.92	0.93	0.45	0.31	0.26	…	0.27	0.60
	RG	0.95	0.96	0.76	0.96	0.96	0.97	0.94	0.87	0.89	0.94	0.92	0.87	0.43	…	0.96	0.80
DSS	MAKM	0.09	0.01	0.75	0.67	0.38	0.03	0.03	0.37	0.44	0.74	0.09	0.34	0.90	…	0.92	0.45
	U-NET	0.98	0.96	0.56	0.97	0.97	0.86	0.76	0.40	0.96	0.96	0.62	0.47	0.42	…	0.43	0.69
	RG	95.3	93.9	83.5	92.1	94.5	96.2	92.2	82.0	79.8	91.2	92.9	46.8	26.8	…	76.7	71.1
Sn (%)	MAKM	18.2	3.5	89.1	99.3	100.0	7.4	100.0	96.9	99.4	99.9	25.8	98.5	82.4	…	85.5	89.6
	U-NET	98.9	97.7	85.7	98.0	98.8	97.2	60.9	98.3	92.9	95.4	45.2	99.9	89.4	…	97.8	90.7
	RG	99.8	100.0	98.2	100.0	99.9	99.9	100.0	99.8	100.0	99.8	99.8	100.0	100	…	100	99.8
Sp (%)	MAKM	87.9	90.9	97.6	95.7	84.7	82.9	76.8	87.4	83.3	95.3	91.6	83.7	100	…	100	88.6
	U-NET	99.8	99.9	93.7	99.8	99.8	98.8	100.0	88.9	99.9	99.8	100.0	85.8	83.5	…	75.7	92.1
	RG	0.05	0.02	0.35	0.01	0.03	0.02	0.03	0.06	0.00	0.03	0.09	0.00	0	…	0	0.06
EF	MAKM	0.18	0.03	0.89	0.99	1.00	0.07	1.00	0.97	0.99	1.00	0.26	0.98	0.82	…	0.85	0.90
	U-NET	0.99	0.98	0.86	0.98	0.99	0.97	0.61	0.98	0.93	0.95	0.45	1.00	0.89	…	0.98	0.91
	RG	0.95	0.94	0.83	0.92	0.94	0.96	0.92	0.82	0.80	0.91	0.93	0.47	0.27	…	0.77	0.71
OF	MAKM	0.18	0.03	0.89	0.99	1.00	0.07	1.00	0.97	0.99	1.00	0.26	0.98	0.82	…	0.85	0.90
	U-NET	0.99	0.98	0.86	0.98	0.99	0.97	0.61	0.98	0.93	0.95	0.45	1.00	0.89	…	0.98	0.91
	RG	165.52	174.19	147.51	167.26	166.43	170.25	188.41	157.89	154.39	159.74	169.80	145.23	141.3	…	149.8	157.0
PNSR	MAKM	129.72	132.99	146.42	142.69	130.06	126.75	125.49	131.81	129.36	142.02	134.30	129.55	155.0	…	154.1	138.6
	U-NET	172.31	175.68	137.87	170.98	171.49	154.11	175.68	133.12	163.80	164.69	157.43	130.96	129.1	…	125.8	152.0

**Table 5:** Comparison of the proposed approach with U-Net and its variants using BRATS2015 dataset.

Authors, Year and Citation	Model	Dataset	DSS
Daimary et al. [42]	U-SegNet	BRATS2015	0.73
Zhou et al., 2019	OM-Net + CGAp	BRATS2015	0.87
Kayalibay et al., 2017	CNN + 3D filters	BRATS2015	0.85
Isensee et al., 2018	U-Net + more filters	BRATS2015	0.85
	+ data augmentation		
	+ dice-loss		
Kamnitsas et al., 2016	3D CNN + CRF	BRATS2015	0.85
Qin et al., 2018	AFN-6	BRATS2015	0.84
Havaei et al. [43]	CNN(whole)	BRATS2015	0.88
Havaei et al. [43]	CNN(core)	BRATS2015	0.79
Havaei et al. [43]	CNN(enhanced)	BRATS2015	0.73
Pereira et al. [44]	CNN(whole)	BRATS2015	0.87
Pereira et al. [44]	CNN(core)	BRATS2015	0.73
Pereira et al. [44]	CNN(enhanced)	BRATS2015	0.68
Malmi et al. [45]	CNN(whole)	BRATS2015	0.80
Malmi et al. [45]	CNN(core)	BRATS2015	0.71
Malmi et al. [45]	CNN(enhanced)	BRATS2015	0.64
Taye et al., 2018 [46]	MAKM	BRATS2015	0.68
Re-implemented	U-Net	BRATS2015	0.75
Erena et al., 2020	Case-1:Proposed Approach (15 randomly selected images)	BRATS2015	0.89
Erena et al., 2020	Case-2:Proposed Approach (12 randomly selected images)	BRATS2015	0.90
Erena et al., 2020	Case-3:Proposed Approach (800 brain images)	BRATS2015	0.80
Erena et al., 2020	Average:Proposed Approach	BRATS2015	0.86
